# Mitochondrial Dysfunction and the Aging Immune System

**DOI:** 10.3390/biology8020026

**Published:** 2019-05-11

**Authors:** Peter J. McGuire

**Affiliations:** Metabolism, Infection and Immunity Section, National Human Genome Research Institute, National Institutes of Health, Bethesda, MD 20892, USA; peter.mcguire@nih.gov; Tel.: +1-301-451-7716

**Keywords:** aging, mitochondria, inflammation, innate immunity, adaptive immunity, immunosenescence

## Abstract

Mitochondria are ancient organelles that have co-evolved with their cellular hosts, developing a mutually beneficial arrangement. In addition to making energy, mitochondria are multifaceted, being involved in heat production, calcium storage, apoptosis, cell signaling, biosynthesis, and aging. Many of these mitochondrial functions decline with age, and are the basis for many diseases of aging. Despite the vast amount of research dedicated to this subject, the relationship between aging mitochondria and immune function is largely absent from the literature. In this review, three main issues facing the aging immune system are discussed: (1) inflamm-aging; (2) susceptibility to infection and (3) declining T-cell function. These issues are re-evaluated using the lens of mitochondrial dysfunction with aging. With the recent expansion of numerous profiling technologies, there has been a resurgence of interest in the role of metabolism in immunity, with mitochondria taking center stage. Building upon this recent accumulation of knowledge in immunometabolism, this review will advance the hypothesis that the decline in immunity and associated pathologies are partially related to the natural progression of mitochondrial dysfunction with aging.

## 1. Introduction

The ancestry of the mitochondrion originated ~2.5 billion years ago within the bacterial phylum α-Proteobacteria, during the rise of eukaryotes [1]. The endosymbiotic theory, advanced with microbial evidence by Dr. Lynn Margulis in the 1960s, proposed that one prokaryote engulfed another resulting in a quid pro quo arrangement and survival advantage [2]. The ability of mitochondria to convert organic molecules from the environment to energy led to the persistence of this pact.

Since most cells contain mitochondria, the clinical effects of mitochondrial dysfunction are potentially multisystemic, and involve organs with large energy requirements [3]. In addition to making energy, the basis of life, mitochondria are also involved in heat production, calcium storage, apoptosis, cell signaling, biosynthesis, and aging—all important for cell survival and function [4,5,6,7]. A decline in mitochondrial function and oxidant production has been connected to normal aging and with the development of a variety of diseases of aging. These topics are explored more thoroughly in other articles in this special edition. While the human immune system undergoes dramatic changes during aging, eventually progressing to immunosenescence [8], the role of mitochondrial dysfunction in this process remains largely absent in the literature. Consequently, the purpose of this review is to highlight three important issues in the aging immune system: (1) inflammation with aging; (2) susceptibility to viral infections; (3) impaired T-cell immunity. These clinical phenotypes will be related to our current knowledge on the role of the mitochondria in immune function. As the associations discussed are largely speculative, it is hoped that this review will serve as a stimulus for further investigation into these issues.

## 2. Is There a Mitochondrial Etiology for “Inflamm-Aging”?

The term “inflamm-aging” (IA) refers to a low-grade, chronic inflammatory state that can be found in the elderly [9]. IA increases morbidity and mortality in older adults, and nearly all diseases of aging share an inflammatory pathogenesis including Alzheimer’s disease, atherosclerosis, heart disease, type II diabetes, and cancer [9]. Nevertheless, the precise etiology of IA and its causal role in contributing to adverse health outcomes remain largely unknown.

**Figure 1:**
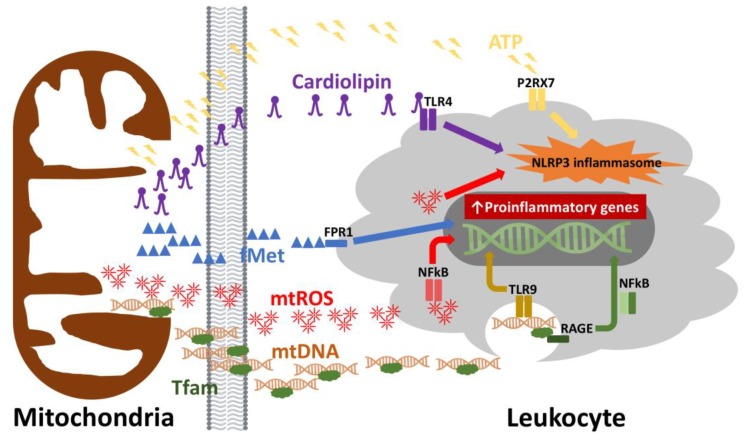
Mitochondrial damage associated molecular patterns (DAMPs). DAMPs derived from mitochondrial components may be released during cellular injury, apoptosis or necrosis. Once these mitochondrial components are released into the extracellular space, they can lead to the activation of innate and adaptive immune cells. The recognition of mitochondrial DAMPs involves toll-like receptors (TLR), formyl peptide receptors (FPR) and purigenic receptors (P2RX7). By binding their cognate ligands or by direct interaction (i.e., reactive oxygen species, ROS), intracellular signaling pathways such as NFkB and the NLRP3 inflammasome become activated resulting in a proinflammatory response. TLR4 = toll-like receptor 4, TLR9 = toll-like receptor 9, P2RX7 = purigenic receptor, FPR1 = formyl peptide receptor 1, NLRP3 = NLR Family Pyrin Domain Containing 3, fMet = N-formylmethionine, mtROS = mitochondrial reactive oxygen species, mtDNA = mitochondrial DNA, Tfam = transcription factor A, mitochondrial, RAGE = receptors for advanced glycation end-products, NFkB = nuclear factor kappa-light-chain-enhancer of activated B cells.

The ability of the innate system to respond to a wide variety of pathogens lies in germline-encoded receptors, whose recognition is based on repetitive molecular signatures. These pattern recognition receptors (PRRs) are present on the cell surface and intracellular compartments. Toll-like receptors (TLRs), retinoic acid-inducible gene I-like receptors (RLRs), nucleotide oligomerization domain-like receptors (NLRs) and cytosolic DNA sensors (cGAS and STING) are prime examples [10]. Ligands for these receptor systems comprise pathogen associated molecular patterns (PAMPs) and damage associated molecular patterns (DAMPs) [11]. PAMPs are derived from components of microorganisms and are recognized by innate immune cells bearing PRRs. In contrast to PAMPs, DAMPs are endogenous “danger signals” that are released by cells during stress, apoptosis or necrosis. DAMPs can arise from a variety of components normally sequestered to the mitochondria, when upon release, induce inflammation via recognition by the same PRRs that recognize PAMPs [12,13]. Events downstream of PRR engagement include caspase-1 activation with the release of pro-inflammatory cytokines [14]. Examples of mitochondrial DAMPs (mtDAMPs) include cardiolipin, n-formyl peptides (e.g., fMet), mitochondrial transcription factor A (TFAM), adenosine triphosphate (ATP), reactive oxygen species (mtROS), and mitochondrial DNA (mtDNA) (Figure 1). From an evolutionary standpoint, select mitochondrial products produce inflammation due to their prokaryotic origins: e.g., cardiolipin (TLR), fMet (formyl peptide receptor 1, FPR1), and mtDNA (TLR, NLR, cGAS) [15,16,17,18,19,20,21,22]. However, mtDAMPs are not just limited to bacterial mimics. TFAM, a nuclear gene and key regulator of mtDNA transcription and replication, activates immune cells via receptors for advanced glycation end products (RAGE) and TLR9 [23,24]. Products of oxidative phosphorylation (OXPHOS) can also stimulate innate immune cells. Released from apoptotic or necrotic cells, ATP binds to purigenic receptors initiating inflammation [25], while mtROS modifies core immune signaling pathways involving hypoxia inducible factor 1 alpha (HIF1α) and nuclear factor kappa light chain enhancer of activated B-cells (NFkB) [26,27].

mtDAMPs contribute to a host of inflammatory diseases, including sepsis, systemic inflammatory response syndrome (SIRS), ischemic reperfusion injury, and aging [28]. One of the consequences of failing mitochondria due to aging, beyond mtROS, is the release of mtDNA. Plasma levels of mtDNA increase gradually after the fifth decade of life, correlating with elevated levels of pro-inflammatory cytokines (i.e., TNF-α, IL-6, RANTES, and IL-1ra) [29]. These data indicate that mtDNA may promote the production of pro-inflammatory cytokines in aging. Because cell stress, senescence and death are a part of the pathophysiology of aging [30], designing new therapeutic strategies against circulating mtDNA, or other mtDAMPs, or their cognate receptors (e.g., TLRs or FPR1) may be a viable strategy to approaching IA and its associated conditions.

## 3. Is Increased Susceptibility to Viral Infections Related to Depressed Mitochondrial Anti-Viral Signaling Pathways?

In general, older adults are more susceptible to a variety of viral infections, especially respiratory viral infections, resulting in high morbidity and mortality. For example, adults over the age of 65 exhibit a vulnerability to influenza A virus (IAV), and account for ≥90% of IAV-related deaths annually [31,32]. Type I interferons (e.g., IFN-α and IFN-β) are essential cytokines involved in the host antiviral response. Secreted by numerous cell types such as lymphocytes, monocytes, macrophages, dendritic cells, fibroblasts, endothelial cells, osteoblasts and others, type I interferons: (1) limit viral spread by inducing antiviral states in infected and neighboring cells; (2) stimulate antigen presentation and natural killer cell function; and (3) promote antigen-specific T and B cell responses and immunological memory. Interestingly, mitochondria play a major part in innate immune signaling against viruses and the production of type I interferons and will be discussed further.

**Figure 2:**
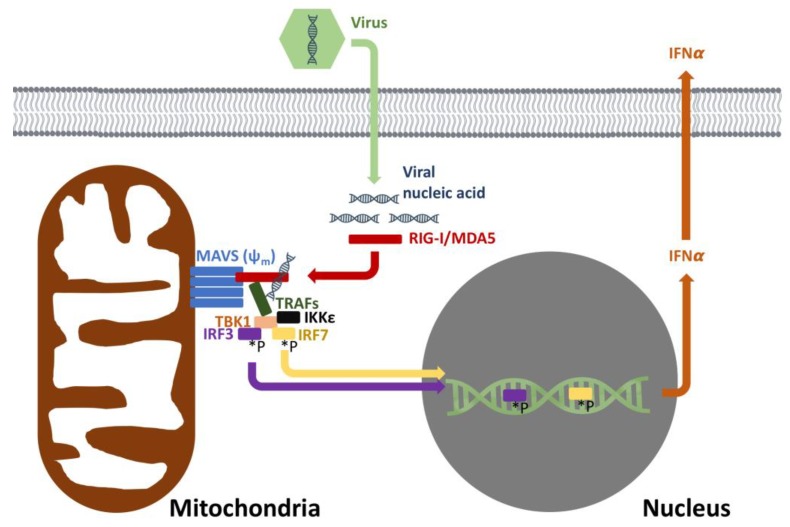
Mitochondrial antiviral response. The recognition of viral nucleic acids involves mitochondria and intact membrane potential (ψ_m_). RIG-I and MDA5 recognize cytoplasmic viral nucleic acids, leading to the oligomerization of MAVS. MAVS then sets in motion a signaling pathway that eventually leads to the phosphorylation (*P) of IRF3/7 with subsequent induction of IFNα to offer antiviral cellular protection. RIG-I = retinoic acid inducible gene I, MDA5 = melanoma differentiation-associated protein 5, MAVS = mitochondrial antiviral-signaling protein, TRAFs = TNF receptor associated factors, TBK1 = TANK binding kinase 1, IKKε = inhibitor of nuclear factor kappa-B kinase subunit epsilon, IRF 3 = interferon response factor 3, IRF 7 = interferon response factor 7, INFα = interferon alpha.

RLRs (e.g., RIG-I and MDA5) are cytosolic receptors that recognize viral RNA. Consequent to binding viral RNA, RIG-I and MDA5 mobilize the mitochondrial antiviral signaling protein (MAVS) [33,34]. MAVS is a 56 kDa protein which contains an N-terminal caspase recruitment domain (CARD), a proline-rich region and a C-terminal transmembrane domain. Anchored on the outer membrane of the mitochondria, peroxisomes and mitochondrial associated membranes (e.g., endoplasmic reticulum), MAVS assembles into prion-like aggregates following RIG-I or MDA5 binding (Figure 2). MAVS aggregates serve as a scaffold to recruit various TNF receptor associated factors (TRAFs), resulting in phosphorylation and nuclear translocation of interferon regulatory factors (IRFs) [35]. Downstream of MAVS, IRF3, IRF5 and IRF7 bind to their cognate promoters, leading to the production of type I interferons [36]. The localization of MAVS to the outer mitochondrial membrane is not coincidental. MAVS activity has been found to be dependent upon intact mitochondrial membrane potential, and by extension OXPHOS function [37].

To date, studies addressing MAVS function during aging and its relationship to waning antiviral immunity are lacking. Decreased mitochondrial membrane potential, mitochondrial dysfunction and declining mitophagy occur in a variety of aging cell types [38,39], raising the question of whether MAVS dysfunction can occur due to mitochondrial failure with aging. Mitochondrial respiratory capacity is impaired in aging monocytes [40] as is phosphorylation of IRF3 and IRF7, suggesting a link with MAVS [41]. As a result, type I IFN synthesis is significantly lower in dendritic cells and monocytes from aging individuals [42,43]. In addition to a decline in mitochondrial respiration, oxidative stress, another consequence of aging, may also be involved in this process [43].

## 4. Is Impaired T-Cell Immunity in Aging Related to a Decline in Mitochondrial Function?

Aging-related decline in immune function (i.e., immunosenescence) renders older individuals more vulnerable to infectious diseases and cancer, resulting in increased morbidity and mortality. Besides increased susceptibility to infection, vaccine efficacy is significantly reduced in the elderly, limiting the utility of prophylaxis [44,45]. Undeniably, profound changes in T-cell function are evident in older individuals, and these changes may be related to a decline in mitochondrial function.

T-cells play a central role in the coordination of adaptive immune responses and cell-mediated immunity. The ability of T-cells to fulfill this role is dependent upon rapid cellular proliferation and differentiation. In response to infection, T-cells proliferate every 4–6 h, generating >10^12^ cells in one week [46,47]. This is accompanied by an increase in size, DNA remodeling, up-regulation of transcription factors and effector molecules, and increased expression of surface proteins [48,49], thus necessitating a large metabolic demand. To accomplish this task, metabolic fuels including fats, sugars and amino acids are actively transported across the cell membrane to feed the increase in energetic demands [50,51]. Along with this increased transport, T-cells undergo metabolic reprogramming during their transition from a naïve state to activated and differentiated cell types (e.g., effector, regulatory and memory cells).

The diverse roles played by mitochondria in T-cell activation emphasizes the potential mechanisms by which aging-related mitochondrial decline may contribute to immune dysfunction. Following stimulation of the T-cell receptor, T-cells undergo substantial changes in intermediary metabolism including an increase in glycolysis and OXPHOS [52,53,54,55,56,57]. In the presence of oxygen, pyruvate produced via glycolysis is fully oxidized in the mitochondria for energy in many cell types [58,59]. In T-cells, a significant proportion of glucose is not oxidized, but rather fermented to lactate from pyruvate via lactate dehydrogenase. This is done despite the presence of oxygen, and is termed aerobic glycolysis or Warburg metabolism [50,56,60]. Although Warburg metabolism is viewed as energetically inefficient, the rate of glycolysis is 10–100 times faster than glucose oxidation by the mitochondria, yielding equivalent amounts of ATP [61]. The additional payoff of Warburg metabolism lies in pathways that are branch points off of glycolysis (e.g., pentose phosphate pathway) which yield reducing equivalents for biosynthesis and nucleotides. Despite this adoption of the Warburg phenotype, OXPHOS is still required for T-cell activation [57]. ATP derived from the mitochondrial respiration promotes enhanced glycolysis as well as the initiation of proliferation in activated T-cells [62]. While pyruvate is mostly diverted to lactate rather than acetyl-CoA via pyruvate dehydrogenase, TCA function and the generation of reducing equivalents in highly proliferating cells is still maintained through anapleurosis: glutamine is converted to α-ketoglutarate via glutaminolysis [63,64]. Bioenergetic studies of aging tissues are consistent with a progressive decline in mitochondrial respiratory function due to a decrease in respiratory complex activity, mitochondrial membrane potential, and impaired mitophagy [39,65]. As a result, impaired OXPHOS results in reduced ATP production, thus potentially limiting glycolysis, biosynthesis and the attainment of biomass during T-cell activation and proliferation.

Besides engaging in bioenergetics, mitochondria also function in T-cell activation by modulating secondary messengers including calcium (Ca^2+^) and reactive oxygen species (ROS). In activated T-cells, mitochondria localize to the immune synapse, and where they regulate Ca^2+^ flux [5,6]. In response to this calcium flux, ROS production via complex III of the respiratory chain is amplified, leading to nuclear factor of activated T-cells (NFAT) activation and subsequent interleukin-2 (IL-2) production [66]. Aged T-cells show reduced Ca^2+^ signaling, which could be partly due to deficits in Ca^2+^ regulation found in mitochondria of aged cells [67,68], theoretically yielding perturbations at the immune synapse causing diminished T-cell signaling and activation.

**Figure 3:**
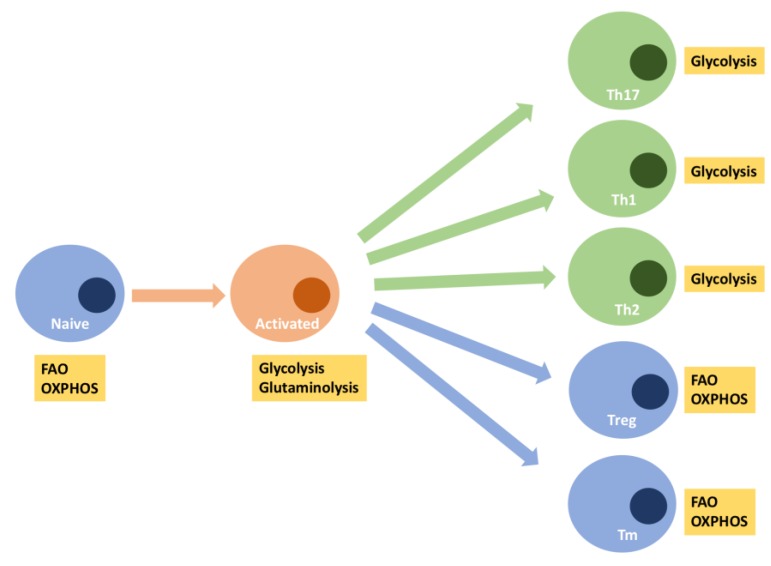
T-cell activation and differentiation involved metabolic reprogramming. At rest, naïve T-cells primarily use OXPHOS to derive their energy. Following activation, T-cells switch to Warburg metabolism and glutaminolysis to support their proliferative needs. Differentiation into T-helper subsets can involve either the maintenance of the Warburg phenotype (i.e., Th17, Th1, Th2), or the reversion to OXPHOS with FAO (i.e., Treg, Tm) as an important fuel. FAO = mitochondrial fatty acid oxidation, OXPHOS = oxidative phosphorylation, Th17 = T-helper cell 17, Th1 = T-helper cell 1, Th2 = T-helper cell 2, Treg = regulatory T-cells, Tm = memory T-cells.

Depending on the cytokine milieu, helper T-cells (Th), marked by the surface expression of CD4, differentiate into various effector subsets comprising T-helper 1 (Th1), T-helper 2 (Th2), T-helper 17 (Th17), regulatory T-cells (Treg). Each of these T-cell subsets are unique in their responsibilities and are identified by their cytokine signatures. Accompanying these functional distinctions are differences in metabolic reprogramming (Figure 3). For example, for T-cells subsets involved in inflammation (e.g., Th1 and Th17), the Warburg metabolism instituted at T-cell activation persists [69]. Despite this primary use of glycolysis, intact OXPHOS is still necessary for their function [57]. The effects of mitochondrial dysfunction may be more readily seen in regulatory (Treg) and memory (Tmem) T-cells. Tregs, which serve to modulate the immune system and maintain tolerance, revert back to OXPHOS as their main pathway for generating energy upon differentiation [69]. Tmem follow a similar metabolic path. Tmem are critical for adaptive immune responses characterized by robust responses to secondary immune challenges. Unlike effector T-cells, Tmem do not undergo extensive proliferation and produce little or no cytokines. As such, the metabolic profile of Tmem are essentially catabolic, relying on OXPHOS and fatty acid oxidation [70,71]. Therefore, it is not surprising to find that CD8^+^ cytotoxic memory T-cells have high respiratory capacity and increased mitochondrial mass, which allows them to rapidly reactivate upon re-exposure to their cognate antigens [62,72]. Given the age-related decline in mitochondrial function as described above, T-cell subsets which are critical for immunosurveillance and the clearance of invading pathogens could be functionally impaired and may partially explain the vulnerability to infection and cancer with aging [57]. Emerging data also suggest that aging significantly affects Treg frequencies, subsets and function [73], potentially leading to the increased incidence of autoimmunity, oftentimes seen with aging [74]. As noted above, Tmem also rely heavily on OXPHOS. Therefore, aging-related deficiencies in Tmem may also be traced to declining OXPHOS, manifesting as impaired immune memory to novel antigens and suboptimal boosts to existing memory [75].

## 5. Conclusions

Virtually every country in the world is experiencing the challenges associated with accelerated growth in the aging population. With this graying of the population comes an increased incidence in diseases of aging, many of which have an immune component. As a result, understanding the pathophysiology of diseases of aging is now more important than ever. In this review, three main immune issues prevalent in the aging population were addressed: (1) inflamm-aging; (2) increased vulnerability to infection; and (3) declining T-cell immunity. The role of the mitochondria in inflammation and immunity, combined with the knowledge of a decline in mitochondrial function with aging, has been synthesized in this review in an effort to partially explain the immune phenotype associated with aging. However, further examination of this relationship is needed. As the methods of inquiry into mitochondrial biology continue to expand, so will investigations into the relationship between this ancient organelle and immunity in the aging population.
